# Genetic and family counselling for schizophrenia: Where do we stand now?

**DOI:** 10.4102/sajpsychiatry.v22i1.831

**Published:** 2016-05-06

**Authors:** Johannes L. Roos

**Affiliations:** 1 Department of Psychiatry, University of Pretoria, South Africa

## Abstract

**Background:**

Recent genetic findings have led to profound changes in genetic and family counselling for schizophrenia patients and their families.

**Objectives:**

The article gives an overview of the present knowledge regarding the genetic and family counselling for schizophrenia.

**Method:**

Literature searches were performed on the MEDLINE database (2011–2015) and African Healthline. A current alert service which provides the most recent literature on the topic on a monthly basis was also used in the study. A clinical case example is presented as is experienced in daily psychiatric practice.

**Results:**

Genetic risk communication has become the responsibility of the multiprofessional treatment team, moving away from specialists in the field. The treatment team provides information on a daily basis regarding risk predictors in the management of schizophrenia, including risk of relapse, suicide and comorbid substance use. Although genetic information is unique and has implications for blood relatives, genetic risk factors only rarely provide information that is inherently different from that provided by other risk predictors commonly used in healthcare. The common variant common disease and rare variant common disease models as contrasting hypothesis of the genetics of schizophrenia are discussed and debated. An example of a family counselled is given and the place of commercial companies that offer directly to the consumer affordable personal DNA testing for psychiatric illness is discussed. Ethical issues without resolution regarding genetic counselling of schizophrenia are debated.

**Conclusions:**

Recent genetic findings must lead to profound changes in genetic and family counselling in schizophrenia. Exposed attributable risk has immediate effects on genetic counselling of schizophrenia. Psychiatric risk counselling has thus changed from risk estimates based on family history to estimates based on test results in specific individuals.

## Introduction

A article by the Schizophrenia Working Group of the Psychiatric Genomics Consortium was published in *Nature* in 2014^1^ and was extensively discussed in the media. It was said that many findings in the study have the potential to provide entirely new insights into the aetiology of schizophrenia. Patients and their family members wanted to know how these findings affect their illness.

Recent genetic findings led to profound changes in genetic and family counselling for schizophrenia patients. The shift in responsibility for genetic risk communication away from genetic specialists is a natural transition in medicine for practises once limited to specialists to be adopted by generalists.^2^ The transitions are also driven by a lack of access to and availability of genetic specialists. Currently, there are only 18 registered genetic counsellors (of which 3 are interns) in South Africa (SA). According to the Genetic Service Plan for SA, the suggested genetic counsellor-to-person ratio is 1:500 000, which translates to a recommended > 100 genetic counsellors in SA. At present, there are only two genetic counsellor posts available in state practice in the Western Cape, both of which are part-time contract posts (4 days/week). The Gauteng Province has four permanent posts and KwaZulu-Natal one. In comparison, the United States of America has 2500 registered genetic counsellors.^3^

Although genetic information is unique and has implications for blood relatives, genetic risk factors only rarely provide information that is inherently different from that provided by other risk predictors commonly used in healthcare.^2^ Psychiatrists and generalists provide information on a daily basis regarding risk predictors in the management of schizophrenia, including risk of relapse, suicide and comorbid substance use.

## The genetics of schizophrenia

The genetic architecture for schizophrenia has proven to be complex. The debate on the complexity focuses on the relative merits of two contrasting (but conceptually related) hypothesis: the common variant common disease and rare variant common disease models.

The common variant common disease model proposes that genetic risk in an individual and in the population is attributable to many high-frequent variants, each conferring modest level of risk.^4,5^ By contrast, the rare variant common disease model proposes that genetic risk in an individual can be explained by rare mutations that confer significant risk. Thus, the common disease might reflect a large number (hundreds or thousands) of different causes, have low frequencies (typically less than 1/1000 individuals), but accounting for a large portion of attributable risk in aggregate.^6^

In an expert review, Visscher et al. conclude that the perceived dichotomy between ‘common’ and ‘rare’ variants is not only false but also unhelpful in making progress towards increasing our understanding of the genetic bias of psychiatric disorders.^7^ Strong evidence has been accumulated that is consistent with the contribution of many genes to risk of disease across a wide range of allele frequencies and with a substantial nucleotide polymorphisms (SNPs) on commercial genotyping arrays. At the same time, most causal variants that segregate in the population are likely to be rare, and in total, these variants also explain a significant proportion of genetic variation. It is the combination of allele frequency, effect size and functional characteristics that will determine the success of new experimental paradigms such as whole-exome/genome sequencing to detect such loci. Empirical results suggest that nearly half of genetic variance is tagged by SNPs on commercial genome-wide chips but that individual causal variants have a small effect size, on average. They concluded that larger experimental sample sizes are essential to further our understanding of the biology underlying psychiatric disorders.^7^

The largest molecular genetic study of schizophrenia demonstrated the power of GWAS (Genome-wide association study) to identify large numbers of risk loci^1^ The study group report a multi-stage schizophrenia genome-wide association study of up to 36 989 cases and 113 075 controls. They identified 128 independent associations spanning 108 conservatively defined loci that met genome-wide significance, 83 of which have not been previously reported. Associations were enriched amongst genes expressed in the brain, providing biological plausibility for the findings. Many findings have the potential to provide entirely new insights into aetiology, but associations at DRD2 and several genes involved in glutamatergic neurotransmission highlight molecules of known and potential therapeutic relevance to schizophrenia and are consistent with leading pathophysiological hypotheses.^1^

Independent of genes expressed in the brain, associations were enriched amongst genes expressed in tissue that have important roles in immunity, providing support for the speculated link between the immune system and schizophrenia. The researchers conclude how that variation in the identified genes has an impact on function to increase the risk for schizophrenia, but cannot be answered by genetics. The overlap strongly suggests that common and rare variant studies are complementary rather than antagonistic and that mechanistic studies driven by rare genetic variation will be informative for schizophrenia.^1^

## What do patients, their families and clinicians want and understand?

**FIGURE 1:**
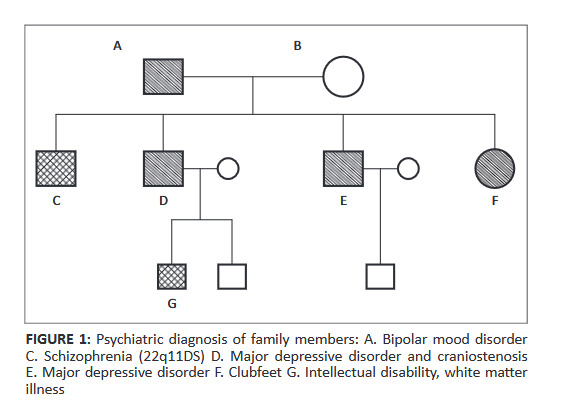
Psychiatric diagnosis of family members: A. Bipolar mood disorder C. Schizophrenia (22q11DS) D. Major depressive disorder and craniostenosis E. Major depressive disorder F. Clubfeet G. Intellectual disability, white matter illness

In a case study, a family was counselled that was included in the ongoing study of genetics of schizophrenia in the Afrikaner founder population. As part of the study, the following disorders were diagnosed in the family (Figure 1).

The family can be described as a family with a syndrome of unknown cause.

Mr. E approached the researcher regarding having further children because of the strong family history of psychiatric illness and his brother’s son having intellectual disability (ID). Patient C was hospitalized because of treatment-resistant schizophrenia. Parents (A + B) also came for a consultation regarding placement of Patient C. It was possible to have whole-genome sequencing performed on all family members because this may be included as a family with a syndrome of unknown cause. An ethical issue arose during the interview with the parents (A + B) before the information can be conveyed to the family.

The father Mr. A, being a professional person, has performed extensive research on the Internet and was aware of de novo mutation in schizophrenia and other relevant issues. His youngest daughter Ms. F was planning to marry soon and was not aware of the genetic testing performed on all family members. The brother D, who had a child with ID, was not interested in further genetic testing. The father said brother E may request the testing, as long as the other family members are not involved with the results of the testing.

It is now possible to sequence the individual human genome and detect single-nucleotide variations, microdeletions and duplications within it. Commercial companies have sprung up in a similar manner to the software or electronic industries and have begun to market directly to the consumer DNA testing. Quite often, individuals become involved with these companies to satisfy curiosity about their ancestry. Additionally, commercially available results that appear incidentally can also be distributed to the consumer.

Commercial companies include *23 andme, Navigenics* and *Psynomics*. They offer directly to the consumer affordable personal DNA testing for medical and psychiatric illnesses. There are no government regulations or rules for overseeing this new industry. Results include information on the carrier status for Mendelian diseases, health risks and drug responses. Incidental findings are returned to them as well. It is left to the consumer to understand the implications of their received DNA report and be able to use the resultant risk scores wisely. Some people will approach their physicians whilst others will deal with the information themselves. Decisions may ultimately lead to life-changing decisions on the basis of these reports.^8^

At the onset of a genetic counselling session, it is critical to know what the patient or family wants to obtain from the genetic testing, what knowledge do they have and how informed are they. It is important to ascertain what their expectations from the test results are.

It has become apparent to researchers working on the genetics of schizophrenia that it was a complex non-Mendelian disorder. There are clear differences in opinions between consumers, their clinicians and researchers. These differences could be based on a lack of understanding of the amount of risk conferred on family members by reported gene variants.^8^ Risk for schizophrenia is more than simply a numeric calculation. It is rather a perception of what will be experienced and how severe it will be as well^9^ Most people going to seek such risk information are doing so in order to make reproductive decisions as with Mr. E in the clinical example.^10^ How risk is communicated has substantial impact on an individual’s life decisions and how it is communicated about complex disorders such as schizophrenia is often difficult.^2^ As with Ms. F in the clinical example, her future husband may decide not to continue with the marriage. Mr. E’s wife may blame him and his family for the risk of psychiatric illness in their son and future siblings.

The genetic architecture of schizophrenia is still unknown, and there is still no proven genetic risk factor consistently replicated in independent studies that confer risk for schizophrenia, and even if there were, this risk is likely to be so low that a test using it would not be useful at all. One cannot assume that risk is synonymous with prediction, and thus, it is able to determine who will become ill. Risk factors only elevate one’s chances of becoming ill.

There are two generally investigated large major sets of risk factors:

- the common alleles that appear to confer very small risk above the general population,
- the rare CNVs that have high risks for conferring disease

CNVs that exist in the genome are either de novo or transmitted to offspring. CNVs are rare but when pathogenic (i.e. occurring in a functional portion of a gene) are likely to have high risks for conferring disease. Several have been found, such as the very large CNV on chromosome 22q11 (as in Patient C in the clinical case) and one on chromosome 7q36.3. Most of the CNVs associated with schizophrenia have been identified in large studies of unrelated patients and not yet proven to be causative of the disorder with families. They also tend to be more likely de novo spontaneous mutations rather than run in families.^11^

## Ethical issues without resolution

### Incidental findings

Whole-genome sequencing leads to a large accumulation of data. If a company screens for one disease, that is, schizophrenia as in the case discussion, should they be obligated to inform the individual that they also have the proven risk factors for other diseases such as breast cancer or Alzheimer’s disease, if they did not request it? Controversies continue about what should be performed with these findings. The American College of Medical Genetics has released recommendations for 24 conditions that should systematically be disclosed to the patient. At this stage, these are the only clinically useful ones that can lead to benefit to the patient and thus the only ‘incidental’ findings that should be communicated. They do not include genetic risk variation for schizophrenia.^12^

### Should a child be tested for the risk of schizophrenia?

Should a child have a right to choose such a test when he or she is within the age of risk for the illness? In a situation where the child with ID develops psychotic symptoms in his teenage years, should he be tested for risk of schizophrenia? Some believe that if there is no clear premorbid treatment, there is no clinical benefit and thus informing the child is not ethically sound. On the contrary, if the child is symptomatic in a prodromal stage, one might think sharing this risk would be helpful. The danger is of false negatives and thus a false sense of confidence that medication and other preventive measures may not be necessary.^8^

### Should risk of schizophrenia be considered in antenatal or prenatal screening?

If one of the siblings (C – E) in the case study has an inherited 22q11.2 CNV and the couple becomes pregnant, will antenatal or prenatal screening be indicated?

The clearest, yet rare, DNA risk factors that may be worth screening for to date are CNVs that have been shown to be associated with schizophrenia such as the deletion in 22q11.21 that confers an approximately 68% risk of schizophrenia on people who have it. Although one would not put much value in tests with effect sizes too small for clinical utility, in the future aggregate risk factors for a polygenetic component may become usable in genetic counselling. The ethical use of these factors needs to be considered, such as whether either pregnancy planning or pregnancy termination should be considered based on parental or antenatal genetic risk profiling or even pre-implantation in vitro selection of embryos.^8^

Families should be informed that heritability, as an index of genetic influence, may have limited explanatory powers unless viewed within the context of interaction with social effects. The onset of schizophrenia is often associated with environmental factors such as early-life adversity, growing up in an urban environment, minority group position and cannabis use. Longitudinal research is needed to uncover gene–environment interplay. It must be determined how expression on vulnerability in the general population may give rise to more severe psychopathology.^13^

### Should family members have the right to know risk to other relatives?

If an individual is known to be carrying a risk factor for a Mendelian disorder, there may be an issue as to whether it is one’s duty to inform siblings and other close relatives. It is much less clear for risk factors for schizophrenia, as, whilst CNVs could be highly penetrant, they could also have arisen de novo and not have been passed on from a parent, thus suggesting that other relatives may not be at high risk.^8^

### Will genetic testing have a role in marital choice?

Should genetic testing become commonplace in the future. Prospective mates will request DNA results. Where DNA screening differs from syphilis or AIDS screening is that one is then no longer concerned about jeopardizing one’s own health, but rather that of a child that is conceived in the future. It is clear that public ethical debates about DNA testing have not yet reached the forefront of the medical industry worldwide and that, until they do, testing will occur without knowledge of how much damage or good it has done.^8^

### The danger of misused genetic information

In the Hadamar psychiatric facility near Frankfurt in Germany, thousands of patients with schizophrenia, as identified by their psychiatrists, were euthanized in the mistaken belief this could help terminate new generations of offspring who had inherited the illness.^14^ This happened because of a severe lack of understanding of the genetic research findings and how they could be put to practical use in the clinic. Public discussion has not yet addressed the issue of stigma and thus discrimination because of one’s genetic constitution. Discriminatory practices may occur on several levels including applying for jobs, insurance policies and several other.^8^

### Beneficial and negative effects of genetic testing in general to individuals and their clinicians

Beneficial effects include the following:

- provide confirmation of a suspected diagnosis
- more specific and appropriate treatment
- implications for other family members suspected to be at risk for the illness based on family history
- predict likelihood of response to medications
- satisfy an inherent curiosity about human variation and one’s distant origins
- useful for research purposes^8^

Negative effects include the following:

- could be stigmatising
- lead to discrimination in job selection and advancement
- loss of healthcare benefits
- social discrimination^8^

Questions we are now confronted with are as follows: does DNA testing for risk prediction make sense for schizophrenia and does scientific evidence support its clinical utility? Are the ethical problems too great to overcome and first need to be addressed by government regulations for control of this new booming industry?^8^

## Discussion

Counselling individuals about their offspring’s genetic risk must take into account both the more common but less pathogenic SNP risk and the less common but more pathogenic CNV risk.

Elliot Gershon addressed an audience of patients and families with mental illness some years ago.^15^ He told them that the empirical risk of schizophrenia for children of a healthy sib of a patient with schizophrenia was 3%. A woman said that she heard the same number from Franz Kallmann, the founder of psychiatric genetics, 50 years earlier and had subsequently three children, all of whom developed schizophrenia. She commented that if she had known what she knew now, she never would have had children.

Prof Gershon said we must imagine the same question arising today. If the woman sought counselling for schizophrenia risk before she had children, she, her spouse, her affected sib and their parents might be offered whole-genome screening by GWAS. If the GWAS was unrevealing, the empirical risk estimates would be the same as they were 50 years ago. The woman would be cautioned that we know that there also exist high-risk genetic predisposition and that not all of them may be identified at this point.

If the GWAS results revealed that the sibling has a high-penetrance rare CNV with a risk of schizophrenia and other disorders and that the woman is as well carrier of the same CNV, her prospective children who had the CNV would be at high risk. The risks and choices they now have could be specified to her and her prospective co-parent. The reproductive choices the couple would make in response would depend on their personal ethical orientations and their responses to the family history and test results. Informing their decisions would be a range of reproductive technologies available today that were not available in the past. Pre-implantation screening of embryos would avoid the CNV risk. If the test results were that her ill-sib had the rare CNV and she did not, the sib’s risk (exposed attributable risk) would be mainly attributable to his or her CNV and the woman herself would not face an elevated risk of schizophrenia in her children.^15^

In a counselling situation, the epidemiological concept of ‘exposed attributable risk’ is potentially useful. This is the proportion of an individual’s risk that is accounted for by a specific risk factor that he or she has. Exposed attributable risk ranges up to 98% for the 22q11 deletion CNV. The ‘exposed attributable risk’ because of any de novo CNV is 79% in bipolar mood disorder (BMD), 84.1% in schizophrenia and 86.7% in autism spectrum disorder (ASD).^15^ For an affected person with a CNV event to be known to be associated with one or more of these diseases, most of that person’s risk is because of the CNV, with a corresponding reduction in risk for relatives without the CNV. Exposed attributable risk thus has immediate effects on genetic counselling in three disorders considered here, for the non-negligible proportion of cases with de novo CNVs or rare associated CNVs.

Consider the family presented earlier in which there is a patient with schizophrenia and no other known individuals with the disorder. His siblings may be concerned about their own risk of illness and that of their offspring. If the schizophrenia patient has a de novo CNV mutation, the risk to the siblings who do not have that CNV mutation would not be appreciably different from the population risk, because 79% of the patients’ attributable risk is because of the CNV. In this instance, the risk of illness in relatives based on their genotypes is comparable to the population risk (1%) and is not the usual risk in sibs (which would be 3%) for schizophrenia.

Karayiorgou discovered in 1995 the first rare CNV associated with schizophrenia, a large CNV with millions of DNA base pairs on chromosome 22.^16^ The rate of individuals with a de novo CNV anywhere in the genome is 4.3% in cases of bipolar disorder and 6.1% in schizophrenia, compared with 0.9% in controls. Taken together, the CNV findings represent an important class of risk factors for bipolar disorder, schizophrenia and ASD and generate a profound change in the genetic counselling for them. From an epidemiological and counselling viewpoint, the major impact on disease risk is from de novo CNVs.^15^

In the case study, the family member’s rights to genetic information and conflicts and stigma within the family come to the fore. For the practicing mental health professional, narcissistic injury and within-family conflicts of this kind are not unusual psychotherapeutic challenges. The overt content of genetic test results is particular to the counselling offered on risk, but the psychodynamic and interpersonal issues that arise are not unlike other responses to misfortune and illness and the treatment is not different either. Counselling based on genetic tests is new to mental disorders and the particular stigma of mental disorders is a unique aspect of this process for the psychiatric disorders, but even this does not present insuperable psychotherapy technical challenges.^17^

For clinicians involved in psychiatric genetic research, the current ethically acceptable practice in the United States is that individuals can provide information about the health of family members, but only that individuals can retain identifying information or contact the relatives and obtain consent to be tested. This preserves the privacy of the relatives, but greatly impairs the effectiveness of studying extended families, as many investigators who have experience with pedigree studies know.^15^

Recent genetic findings must lead to profound changes in genetic and family counselling. Counselling individuals about their offspring’s genetic risk must take into account both the more common but less pathogenic SNP risk and less common but more pathogenic CNV risk. For associated SNPs in GWAS studies of BMD or schizophrenia, the actual risk of illness to persons who have the risk alleles is small, ranging from 1.01% to 1.10%, compared with the population risk of 1%. This is not a meaningful risk difference to a person receiving genetic counselling. If there is reason to believe that a CNV is involved, the risk calculation changes. In individuals who have do novo CNVs, the risks are 4.3% and 6.1% for BMDs and schizophrenia, respectively, which are considerably greater than the SNP risks, although still modest. For the rare CNVs associated with these disorders, the risks of illness are higher, ranging up to 82% risk that bipolar disorder, schizophrenia or ASDs will develop in a person with the 22q11 deletion. In the aggregate, de novo CNVs are not rare events. If the results of additional studies in BMD and schizophrenia replicate the reported results, one may expect that genotyping of patients and relatives will become a standard procedure for counselling. Screening for CNVs has already become a standard procedure for ASDs, where the data on de novo and rare CNVs are comparable to those for BMD and schizophrenia.^8^

Recent genetic findings must lead to profound changes in genetic and family counselling. A psychotherapeutic approach may be needed as a routine part of risk counselling, particularly for resolution of ethical issues and for within-family stigma and conflicts over genetic results. Psychiatric risk counselling has thus changed from risk estimates based on family history to estimates based on test results in specific individuals.^8^
